# On the Development of Selective Chelators for Cadmium: Synthesis, Structure and Chelating Properties of 3-((5-(trifluoromethyl)-1,3,4-thiadiazol-2-yl)amino)benzo[*d*]isothiazole 1,1-dioxide, a Novel Thiadiazolyl Saccharinate

**DOI:** 10.3390/molecules26061501

**Published:** 2021-03-10

**Authors:** Joana F. Leal, Bruno Guerreiro, Patrícia S. M. Amado, André L. Fernandes, Luísa Barreira, José A. Paixão, Maria L. S. Cristiano

**Affiliations:** 1 Center of Marine Sciences, CCMAR, Gambelas Campus, University of Algarve, UAlg, 8005-139 Faro, Portugal; jfleal@ualg.pt (J.F.L.); brunoecguerreiro@gmail.com (B.G.); patricia.s.amado@gmail.com (P.S.M.A.); alofernandes@ualg.pt (A.L.F.); lbarreir@ualg.pt (L.B.); 2 Department of Chemistry and Pharmacy, Faculty of Sciences and Technology, FCT, Gambelas Campus, University of Algarve, UAlg, 8005-139 Faro, Portugal; 3 CFisUC, Department of Physics, University of Coimbra, 3004-516 Coimbra, Portugal; jap@fis.uc.pt

**Keywords:** cadmium chelation, aquatic remediation, saccharin-based ligands, benzisothiazoles, thiadiazoles, selectivity, molecular structure, π-π stacking

## Abstract

Aquatic contamination by heavy metals is a major concern for the serious negative consequences it has for plants, animals, and humans. Among the most toxic metals, Cd(II) stands out since selective and truly efficient methodologies for its removal are not known. We report a novel multidentate chelating agent comprising the heterocycles thiadiazole and benzisothiazole. 3-((5-(trifluoromethyl)-1,3,4-thiadiazol-2-yl)amino)benzo[*d*]isothiazole 1,1-dioxide (AL14) was synthesized from cheap saccharin and characterized by different techniques, including single crystal X-ray crystallography. Our studies revealed the efficiency and selectivity of AL14 for the chelation of dissolved Cd(II) (as compared to Cu(II) and Fe(II)). Different spectral changes were observed upon the addition of Cd(II) and Cu(II) during UV-Vis titrations, suggesting different complexation interactions with both metals.

## 1. Introduction

Contamination of aquatic systems with heavy metals is a long-standing problem that mostly affects rivers and estuaries where effluents are discharged or to where debris is leached. Several studies point to excessive amounts of metals in the aquatic systems worldwide [1,2], with harmful consequences for aquatic species (e.g., fishes, bivalves, crustaceans, algae [3,4]) and for human health [5,6] as a result of their bioaccumulation and propagation through the food chain.

In this context, the development of effective tools for the selective sequestration of toxic metals is a major challenge. Cadmium is among the toxic metals frequently detected in aquatic systems, mostly arising from the intense industrial, agricultural, and mining activity [7]. Previous studies [8,9,10,11,12,13,14,15,16] focused on adsorption and bioremediation yielded methods that enabled the sequestration of cadmium, but proved unspecific, as other metals were found to be preferentially adsorbed. Thus, the development of specific and effective tools for solving the problem of environmental contamination by cadmium remains a top priority.

**Scheme 1:**
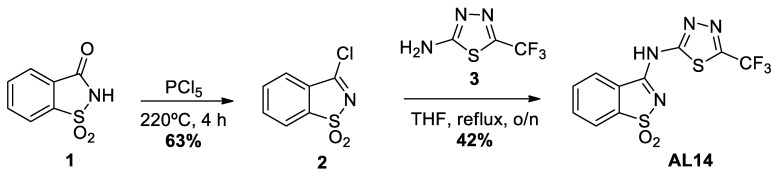
Preparation of 3-((5-(trifluoromethyl)-1,3,4-thiadiazol-2-yl)amino)benzo-[*d*]isothiazole 1,1-dioxide (AL14).

In recent years, we have designed tetrazole-saccharinates [17,18,19,20] and demonstrated their effective and selective chelation capabilities for Cu(II) [17]. It was also demonstrated that the ligands are non-toxic while their corresponding copper complexes proved highly cytotoxic toward several cancer cell lines, suggesting their application in chelant-based antineoplastic chemotherapy. Although useful as tools for selective sequestration of Cu(II), with possible impact in medicine and environmental remediation, tetrazole-saccharinates proved photolabile due to the photoreactivity of the tetrazole moiety [18,21,22]. Optimization of the saccharinate-based conjugates, involving replacement of the partner heterocycle, yielded thiadiazolyl-saccharinates with remarkable properties [19]. In this work, we disclose the synthesis, crystal structure, and chelating properties of *3-((5-(trifluoromethyl)-1,3,4-thiadiazol-2-yl)amino)benzo[d]isothiazole 1,1-dioxide* (AL14; Scheme 1), a novel thiadiazolyl-saccharinate that proved to selectively chelate cadmium from aqueous solution.

## 2. Results and Discussion

### 2.1. Synthesis and Structure Analysis

The preparation of the novel thiadiazolyl saccharinate 3-((5-(trifluoromethyl)-1,3,4-thiadiazol-2-yl)amino)benzo[*d*]isothiazole 1,1-dioxide (AL14; Scheme 1) involved a two-steps synthesis, starting from saccharin (**1**), where the coupling reaction linking the saccharyl (**2**) and thiadiazolyl (**3**) building blocks occurred in moderate yield (Scheme 1).

**Figure 1:**
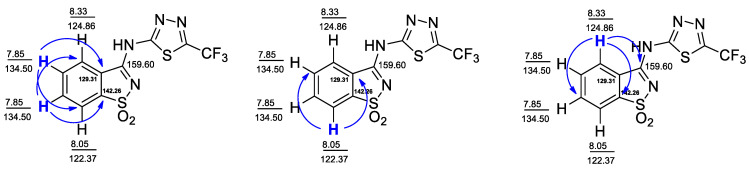
Main HMBC (^3^J) correlations and assignments of signals (ppm) in the ^1^H and ^13^C NMR spectra of compound AL14.

The one-dimensional (1D) and two-dimensional (2D) NMR (Nuclear Magnetic Resonance) data (Supplementary Information, Figures S1–S6) proved the structure of AL14: the four protons of the benzisothiazole moiety can be undoubtedly confirmed by ^1^H NMR (Supplementary Information, Figure S1), following verification by its TOCSY (Total Correlation Spectroscopy) correlations and HSQC (Heteronuclear Single Quantum Coherence) spectra (Supplementary Information, Figures S3 and S4). Additionally, the HMBC (Heteronuclear Multiple Bond Correlation) spectra allowed one to thoroughly identify the carbons of saccharin moiety (Supplementary Information, Figures S5 and S6), enabling better identification of the carbons adjacent to the trifluoromethyl group (Figure 1, Table 1). It is noteworthy that the data reveal the presence of the thiadiazolyl saccharinate in its amino-linked tautomeric form, as also observed in the crystal phase.

### 2.2. X-ray Crystal Analysis

**Figure 2:**
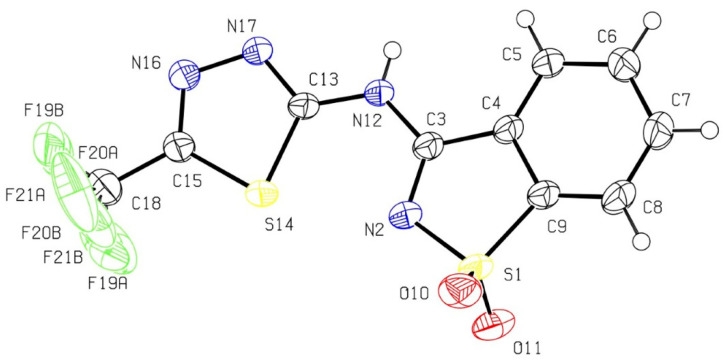
ORTEP (Oak Ridge Thermal Ellipsoid Plot) drawing of the molecule of AL14. The anisotropic displacement ellipsoids are drawn at the 50% probability level.

A single-crystal X-ray crystallography study unambiguously showed that the tautomeric form present in the crystalline state is that depicted in the ORTEP shown in Figure 2 that also depicts the atom labelling scheme. Indeed, all H-atoms could clearly be located from the difference electron density maps. The compound crystallizes in the centrosymmetric triclinic space group *P*−1.

In the crystal, the five- and six-membered rings of the fused benzothiazole group are coplanar, the angle between the least-squares planes of the two rings being 0.57(3)°. The least squares planes of the five-membered thiadiazol-ring and the fused nine-membered ring are closely but not strictly coplanar, the dihedral angle being 7.10(3)°. The molecule has conformational flexibility around the C3—N12 and N12—C13 bonds, but the S14—C13—N12—C3 and C13—N12—C3—N2 torsion angles observed in the crystal are small, being −5.4(2)° and 3.7(2)°, respectively. Overall, the molecule skeleton is closer to planarity as compared to the sulfanyl-bridged thiadiazolyl-saccharinate reported in [19]. The CF_3_ group is disordered over two alternate positions.

Bond lengths and valency angles in the molecule are within typical average values. The N16—C15 (1.288(2) Å), N17—C13 (1.305(2) Å) and N2—C3 (1.298(2) Å) are significantly shorter than N12—C13 (1.372(3) Å) and N12—C3 (1.349(2) Å), showing that the former three bonds have considerable double-bond character. The two S—O bonds have equal lengths (1.422(1) Å). There is a short intermolecular contact between atoms S14 and N2 as the S14—N2 distance (2.651(2) Å) is considerably shorter than the sum of the atoms van der Walls radii (3.35 Å) and even shorter than a similar short contact observed in the crystallographic study reported for the closely related sulfanyl-bridged compound [19].

**Figure 3:**
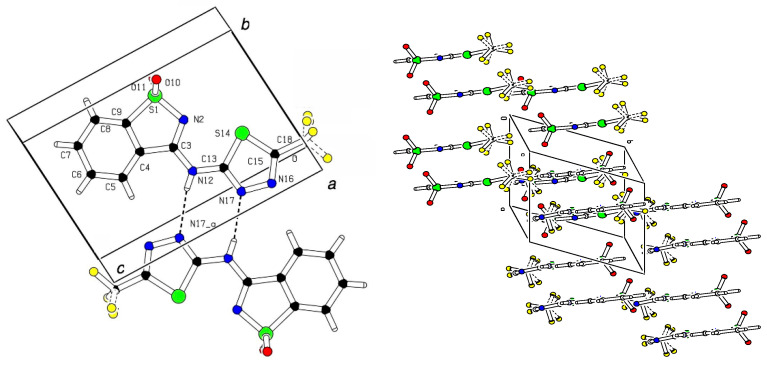
Synthon consisting of a pair of molecules related by an inversion centre strongly hydrogen-bonded via an N—H····N interaction (**left panel**) and crystal packing as a stack of parallel layers of molecules (**right panel**).

The main synthon occurring in the crystal is a pair of molecules, related by an inversion centre, hydrogen-bonded via the interaction N12—H····N17*^i^* (*i* = 1 − *x*, −*y*, 1 − *z*; D—H: 0.80(2) Å; H····A: 2.23(2) Å; D····A: 3.024(2) Å; ∠ D—H—A: 169(2)°) (Figure 3). In addition to this hydrogen bond, two additional intermolecular short contacts could be considered as weak hydrogen bonds, C5—H5····N16^i^ (*i* = 1−*x*,−*y*,1−*z*; D····A: 3.276(2) Å; ∠ D—H—A: 169°) and C7—H7····F19B^ii^ (*ii* = −1+*x*,1+*y*,1+*z*; D····A: 3.341(1) Å; ∠ D—H—A: 144°). The latter connects hydrogen-bonded dimers in parallel layers that are stacked in the structure (Figure 3). Cohesion of the crystal structure likely has a major energetic contribution from π-π interactions between the electron clouds of the rings, as the parallel stacking of layers of molecules positions the rings in a favourable geometry for such interactions. The packing is compact and there are no sizeable voids in the crystal structure accessible to solvent molecules.

### 2.3. Metal Chelating Activities

Driven by the chelating properties evidenced by previously developed saccharin-based conjugates, the chelating activity of 3-((5-(trifluoromethyl)-1,3,4-thiadiazol-2-yl)amino)benzo[*d*]isothiazole 1,1-dioxide (AL14) was first evaluated for Cd(II), Cu(II), and Fe(II), dissolved in water, at different concentrations (1, 5, 10 mM). The results presented in Table 2 reveal a considerably higher chelating activity of AL14 for Cd(II) than for Cu(II) and undetected activity for Fe(II). The results are very important, since most of the studies reporting methodologies to remove Cd(II) from aquatic system are non-selective or preferentially remove other metal ions over cadmium [8,10,12,13,14,15,16]. The number of studies focusing on the specific sequestration of Cd(II) from water is very small [23,24] and a direct comparison with our results is difficult since different experimental conditions were used.

These results clearly show that the novel compound AL14 may be able to selectively chelate Cd(II), compared to Cu(II) and Fe(II). This evidence is reinforced by the fact that the ratio between the ligand and the available metal is lower for Cd(II) than for Cu(II) and Fe(II). A higher ratio of ligand to metal has been associated with greater efficiency of the ligand, as a result of the higher number of binding sites available [14].

**Figure 4:**
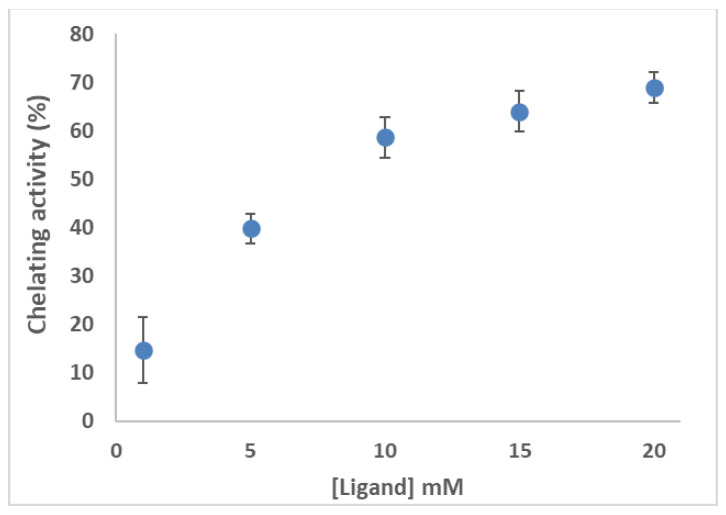
Chelating activity for Cd(II) at different concentrations (1–20 mM).

Since the ligand revealed a greater chelating activity for Cd(II) compared to Cu(II) and Fe(II), additional experiments were developed, now considering higher concentrations (15 and 20 mM) of ligand (AL14). The results show an increase in chelating activity with increasing concentrations, but this increase appears to be slower than that observed in the first experiments at lower concentrations (1–10 mM), suggesting that the equilibrium is being reached (Figure 4). For this set of experiments, the highest chelating activity achieved was 69.0 ± 3.3% for [ligand] = 20 mM and [Cd] = 9 mM.

In order to enable future comparison with the chelating capacity of other compounds, the IC_50_ value for this novel compound was determined. In this context, the IC_50_ corresponds to the concentration of compound (ligand) capable of removing 50% of the metal concentration in solution. The lower the concentration required to remove 50% of the metal, the greater the chelating capacity of the compound. For this new ligand, the IC_50_ is 7.857 mM, with a confidence interval (95%) ranging between 7.585 and 8.139.

### 2.4. UV-vis Titrations

**Figure 5:**
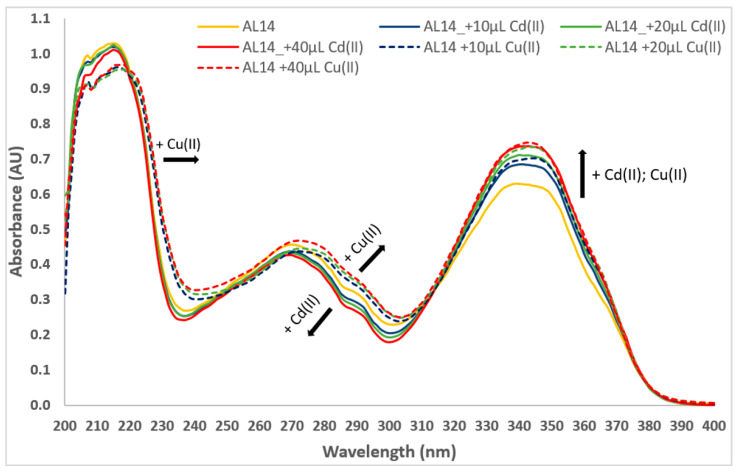
UV-Vis spectra of 3-((5-(trifluoromethyl)-1,3,4-thiadiazol-2-yl)amino)benzo[d]isothiazole 1,1-dioxide (AL14), in ethanol, with different volumes of Cd(II) or Cu(II) addition.

Aiming to evaluate the spectral changes upon metal addition, experiments were performed by adding 2 to 40 µL of aqueous Cd(II) solution (2 mM) to the ligand solution (50 µM, from 1 mM stock solution), in a quartz cuvette. Similar experiments were conducted with Cu(II), for comparative purposes. The spectrum of AL14 (Figure 5) reveals three main bands between 200 and 400 nm. The first and most intense band lies between 200 and 230 nm (at these conditions, its maximum is at ca. 215 nm, *Ɛ* ≈ 2.06 × 10^4^ M^−1^cm^−1^) and it is mainly attributed to saccharin, in keeping with the spectra of other saccharin derivatives also synthesized by our team (data not yet published), and data available in the literature [25,26]. The second and third main bands appear between 240 and 300 nm (*Ɛ*_max_ ≈ 9.14 × 10^3^ M^−1^cm^−1^, at ca. 270 nm), and between 300 and 380 nm (*Ɛ*_max_ ≈ 1.26 × 10^4^ M^−1^cm^−1^, at ca. 339 nm), respectively.

In general, the addition of increasing amounts of Cd(II) promotes an increase in band intensity between 313 and 370 nm, accompanied by a decrease in intensity for wavelengths between 250 and 313 nm, making it possible to identify an isosbestic point at 313 nm (Figure 5). In turn, the addition of the same increasing amounts of Cu(II) causes an increase in the intensity of the third band similar to that observed for Cd(II), which could suggest the same binding pattern [27] for Cd(II) and Cu(II). However, a bathochromic shift of the first and second bands, a decrease in the intensity of the 1st band and an increase in the intensity of the second band are also observed with the addition of Cu(II) (Figure 5), which seems to indicate that the complexation interactions could differ for both metals. Note that for these UV-Vis titration experiments, the molar concentrations of both metals in solution are the same, and the molar concentration of ligand is less than or approximately equal to that of the metals. Thus, under these conditions, only 2:1 (metal: ligand) or 1:1 complexes are expected. Further studies will be conducted to elucidate the structure of the complexes.

## 3. Materials and Methods

### 3.1. Chemicals

All reagents for synthesis were purchased from commercial sources and were used without further purification. Analytical thin layer chromatography (TLC) was carried out using Merck (Darmstadt, Germany) TLC Silica gel 60 F_254_ aluminium sheets and visualized under UV or by appropriate stain. P-anisaldehyde and potassium permanganate were the most used. Column chromatography was carried out using Sigma Aldrich (Darmstadt, Germany) technical grade silica gel (pore size 60 Å, 230–400 mesh particle size, 40–63 μm particle size). In the experiments to determine the chelating ability of the compound, aqueous solutions of buffers, metals, pyrocatechol violet (C_19_H_14_O_7_S, Sigma Aldrich, Darmstadt, Germany), and ferrozine (C_20_H_13_N_4_NaO_6_S_2_, 97.0%, Sigma-Aldrich, Darmstadt, Germany) were prepared using ultrapure water (Simplicity^®^ Water Purification System, Merck, Darmstadt, Germany, 18.2 MΩ.cm). 3CdSO_4_.8H_2_O (98%), CuSO_4_.5H_2_O (p.a, 99%), and FeSO_4_.7H_2_O (p.a, 99%) were purchased from Alfa Aesar, Riedel-de-Haën and Merck (Darmstadt, Germany), respectively. Ligand solution was prepared in dimethyl sulfoxide (99.5%, LAB-SCAN Ltd., Poland). Absolute ethanol (99.8%, Honeywell, Charlotte, NC, USA) was used to prepare the solution of 5,7-Dibromo-8-hydroxyquinoline (97%, Alfa Aesar, Karlsruhe, Germany).

### 3.2. Equipment

^1^H and ^13^C Nuclear Magnetic Resonance (NMR) spectra were recorded using a 400 MHz Bruker (Billerica, MA, USA) instrument or using a 500 MHz JEOL system (Peabody, MA, USA) equipped with a Royal HFX probe in the deuterated solvents described in each experimental procedure. The chemical shifts (δ) are described in parts per million (ppm) downfield from an internal standard of tetramethylsilane (TMS). Melting points (°C) were obtained on an SMP30 melting point apparatus and are uncorrected. High Resolution Mass Spectrometry (HRMS) was recorded using the analytical service within the Centre of Marine Sciences (CCMAR, Algarve, Portugal) and was conducted on a Thermo Scientific High Resolution Mass Spectrometer (HRMS) (Waltham, MA, USA), model Orbitrap Elite, capable of MSn, n up to 10. X-ray diffraction data were collected on a small single-crystal (0.27 × 0.22 × 0.19 mm) at room temperature (at 292 ± 2 K) using a Bruker Apex II diffractometer (Billeriaca, MA, USA) equipped with a 4K CCD detector using graphite monochromated MoKα (λ = 0.71073 Å) radiation. For determination of metal chelating activities, 96-well microplates and a Biotek Synergy 4 Microplate Reader (Winooski, VT, USA) were used. UV-Vis absorption spectra were obtained on a Varian CARY 50 Bio UV-visible spectrophotometer (Varian Inc., Mulgrave, Australia) using quartz cells (1 cm).

### 3.3. Synthesis Procedure

The synthesis of 3-((5-(trifluoromethyl)-1,3,4-thiadiazol-2-yl)amino)benzo[*d*]isothiazole 1,1-dioxide (AL14) was conducted by adapting the methodology developed by Ismael et al. [17,28] (Scheme 1). AL14 was synthesized by coupling 2-amino-5-trifluoromethyl-1,3,4-thiadiazole **3** with *pseudo*-saccharyl chloride **2**, which was previously prepared from commercially available saccharin **1**. Recrystallization from ethyl acetate gave the novel thiadiazolyl saccharinate. Its complete structure elucidation was afforded by ^1^H, ^13^C, and 2D NMR, X-ray crystallography, and HRMS. The standard procedures for its synthesis and the corresponding characterization details are described in the Supporting Information (SI).

### 3.4. X-ray Crystallography

The structure was solved by direct methods using SHELXT-2014/5 [29]. Refinements were carried out with SHELXL-2018/3 [30] by full-matrix least-squares on F^2^, with anisotropic displacement parameters for all non-hydrogen atoms (CCDC 2063726 for details regarding the crystallographic analysis procedures and also for detailed crystal data). All hydrogen atoms could be located on a difference Fourier synthesis; their positions were refined as riding on parent atoms with an isotropic temperature constrained to those of their parent atoms using SHELXL-2018/3 defaults [30], except that of the amino group that is involved in hydrogen bonding, which had its position freely refined with an isotropic displacement factor constrained to 1.2× that of the parent N atom. The CF_3_ group is disordered over two positions with occupancies close to 50%. The final quality factors of the refinement were R_1_ (I > 2σ) = 0.0317, wR_all_ = 0.0921 and GOF = 1.067 for 3097 for independent reflections and 221 refined parameters.

### 3.5. Metal Chelating Activity

3-((5-(trifluoromethyl)-1,3,4-thiadiazol-2-yl)amino)benzo[*d*]isothiazole 1,1-dioxide (AL14) was tested for its Cd(II) chelating activity, at different concentrations (1–20 mM). The chelating activity of AL14 was also assessed in separate experiments towards other potentially competing metal cations (Cu(II) and Fe(II)). The metal chelating activities were evaluated by spectrophotometry, using selective chelating indicators that produce coloured complexes, following methods described in the literature for Cu(II) and Fe(II) [31] and adopting the method described by Ahmed and Chowdhury [32] for Cd(II), with some adjustments for 96-well plates. Thus, the metal ion chelating activities were determined by measuring the colour changes due to the Cd(II)/5,7-dibromo-8-hydroxyquinoline (DBHQ) complex (at 396 nm) [32], Cu(II)/pyrocatechol violet (PV) complex (at 632 nm), and Fe(II)/ferrozine complex (at 562 nm) [17]. The capacity of the tested compound to chelate the metals should be reflected in a decrease in absorbance at each corresponding wavelength. The chelating activity was calculated using the Equation (1), where *A_blank_* and *A_sample_* correspond to the absorption of the solution without and with the ligand, respectively.

(1) $$Chelating\;activity\;\left(\%\right)=\frac{A_{blank}-A_{sample}}{A_{blank}}\times 100$$

For Cd(II), ligand solution (1, 5, 10, 15, 20 mM) was added to aqueous CdSO_4_ (9 mM), followed by DBHQ (3 mM) and H_2_SO_4_ (0.5 mM). For Cu(II), ligand solution (1, 5, 10 mM) was added to sodium acetate buffer (50 mM), followed by aqueous CuSO_4_ (0.2 mM) and PV (4 mM). For Fe(II), ligand solution (1, 5, 10 mM) was added to H_2_O, followed by aqueous FeSO_4_ (0.36 mM) and ferrozine (40 mM). In all experiments, negative controls (blanks) and colour controls (without chelating indicators) were performed. At least three independent experiments (six replicates/each) were conducted, for each experimental condition.

### 3.6. Software

Chemical structures were designed using ChemDraw Ultra 12.0. Graphics and statistical analysis were performed using Microsoft Excel and GraphPad Prism 7.0. Bruker APEXIII [33] software package including SAINT V8.38A [34] and SADABS-2016/2 [35] was used for data reduction of the X-ray crystallography work. SHELXT-2014/5 [29] and SHELXL-2018/3 [30] and PLATON (v. 2010420) [36] were used for the structure solution, refinement and structure analysis and drawing of crystallographic figures, respectively.

## 4. Conclusions

In short, this work unveils a new compound derived from saccharin capable of selectively chelating Cd(II), compared to Cu(II) and Fe(II). 3-((5-(trifluoromethyl)-1,3,4-thiadiazol-2-yl)amino)benzo[*d*]isothiazole 1,1-dioxide (AL14) was analyzed in detail and its structure confirmed by 1D NMR (^1^H and ^13^C), 2D NMR (TOCSY, HSQC, and HMBC), X-ray crystallography, HRMS-ESI^+^ and UV-Vis. In these experiments, the maximum chelating activity for Cd(II), 69.0 ± 3.3%, was achieved for [AL14] = 20 mM and [Cd] = 9 mM, without previous contact time. The concentration of ligand capable of removing 50% of Cd(II) concentration in solution (IC_50_) is 7.857 mM. Reduced or no activity for Cu(II) and Fe(II) was observed, respectively. These are the first studies revealing the potential of this compound for selective Cd(II) chelation. Additional studies will be carried out, considering other environmentally relevant metal ions to assess their possible interference with the chelating capacity of this new and relatively low-cost ligand.

## Figures and Tables

**Table 1:** Correlations found in the TOCSY, HSQC, and HMBC spectra of compound AL14.

^1^H, δ (ppm)	^1^H, δ (ppm) TOCSY	HSQC	^13^C, δ (ppm) HMBC
7.86	8.05; 8.33	134.50	122.23; 124.92; 129.31; 134.44; 142.26
8.05	7.86; 8.33	122.37	129.31; 134.20; 142.26
8.33	7.86; 8.05	124.86	129.07; 134.69; 142,26; 159.60

**Table 2:** Chelating activity (%) of AL14 for Cd(II), Cu(II), and Fe(II).

Concentration (mM).	Cd(II)	Cu(II)	Fe(II)
1	14.7 ± 6.7	NA	NA
5	39.8 ± 3.1	17.7 ± 6.2	NA
10	58.7 ± 4.2	29.2 ± 5.8	NA
15	64.0 ± 4.2	-	-
20	69.0 ± 3.3	-	-

NA—no significant activity.

## Data Availability

A CIF file containing supplementary crystallographic data was deposited at the Cambridge Crystallographic Data Centre with reference CCDC 2063726.

## Supplementary Materials

Figure S1: ^1^H NMR spectra of 3-((5-(trifluoromethyl)-1,3,4-thiadiazol-2-yl)amino)benzo[*d*]isothiazole 1,1-dioxide (AL14) in DMSO-*d_6_*, Figure S2: ^13^C NMR spectra of 3-((5-(trifluoromethyl)-1,3,4-thiadiazol-2-yl)amino)benzo[*d*]isothiazole 1,1-dioxide (AL14) in DMSO-*d_6_*, Figure S3: TOCSY spectra of 3-((5-(trifluoromethyl)-1,3,4-thiadiazol-2-yl)amino)benzo[*d*]isothiazole 1,1-dioxide (AL14) in DMSO-*d_6_*, Figure S4: HSQC spectra of 3-((5-(trifluoromethyl)-1,3,4-thiadiazol-2-yl)amino)benzo[*d*]isothiazole 1,1-dioxide (AL14) in DMSO-*d_6_*, Figure S5: HMBC spectra of 3-((5-(trifluoromethyl)-1,3,4-thiadiazol-2-yl)amino)benzo[*d*]isothiazole 1,1-dioxide (AL14) in DMSO-*d_6_*, Figure S6: HMBC (expanded) spectra of 3-((5-(trifluoromethyl)-1,3,4-thiadiazol-2-yl)amino)benzo[*d*]isothiazole 1,1-dioxide (AL14) in DMSO-*d_6_*, Figure S7: Electrospray ionization mass spectrum in positive-ion mode (HRMS-ESI^+^) of 3-((5-(trifluoromethyl)-1,3,4-thiadiazol-2-yl)amino)benzo[d]isothiazole 1,1-dioxide (AL14), X-ray crystallography data, detailed synthesis procedure, Scheme S1: Preparation of 3-((5-(trifluoromethyl)-1,3,4-thiadiazol-2-yl)amino)benzo[*d*]isothiazole 1,1-dioxide (AL14).

## Sample Availability

The samples are available from the authors.
