# Supplementary material for: On the Development of Selective Chelators for Cadmium: Synthesis, Structure and Chelating Properties of 3-((5-(trifluoromethyl)-1,3,4-thiadiazol-2-yl)amino)benzo[d]isothiazole 1,1-dioxide, a Novel Thiadiazolyl Saccharinate
Source: Molecules. 2021 Mar 10;26(6):1501. doi: 10.3390/molecules26061501 (PMC7999908; doi:10.3390/molecules26061501)
Supplement: Supplementary file 1 [file molecules-26-01501-s001.pdf]

Supporting Information

On the development of selective chelators for cadmium; synthesis, structure and chelating properties of 3-((5-(trifluoromethyl)-1,3,4-thiadiazol-2-yl)amino)benzo[*d*]isothiazole 1,1-dioxide, a novel thiadiazolyl saccharinate.

Joana F. Leal [1,2], Bruno Guerreiro [1,2], Patrícia S. M. Amado [1,2], André L. Fernandes [1,2], Luísa Barreira [1,2], José A. Paixão [3], and Maria L. S. Cristiano [1,2,*]

[1] Center of Marine Sciences, CCMAR, Gambelas Campus, University of Algarve, UAlg, Portugal; jfleal@ualg.pt (J.F.L); brunoecguerreiro@gmail.com (B.G.); patricia.s.amado@gmail.com (P.S.M.A); alofernandes@ualg.pt (A.L.F.); lbarreir@ualg.pt (L.B); mcristi@ualg.pt (M.L.S.C.)

[2] Department of Chemistry and Pharmacy, Faculty of Sciences and Technology, FCT, Gambelas Campus, University of Algarve, UAlg, Portugal

[3] CFisUC, Department of Physics, University of Coimbra, P-3004-516, Coimbra, Portugal; jap@fis.uc.pt (J.A.P.);

* Correspondence: mcristi@ualg.pt; Tel.: +351-289-800-953

Table of contents

**1.**	**Structure analysis**	**S2**
1.1	1D and 2D NMR spectra of 3-((5-(trifluoromethyl)-1,3,4-thiadiazol-2-yl)amino)benzo[*d*]isothiazole 1,1-dioxide	S2
1.2	Mass Spectra of 3-((5-(trifluoromethyl)-1,3,4-thiadiazol-2-yl)amino)benzo[*d*]isothiazole 1,1-dioxide	S8
1.3	X-Ray Cristallography	S9
**2.**	**Experimental Methods**	**S11**
**3.**	**References**	**S13**

1. Structure analysis

1.1 1D and 2D NMR of 3-((5-(trifluoromethyl)-1,3,4-thiadiazol-2-yl)amino)benzo[*d*]isothiazole 1,1-dioxide

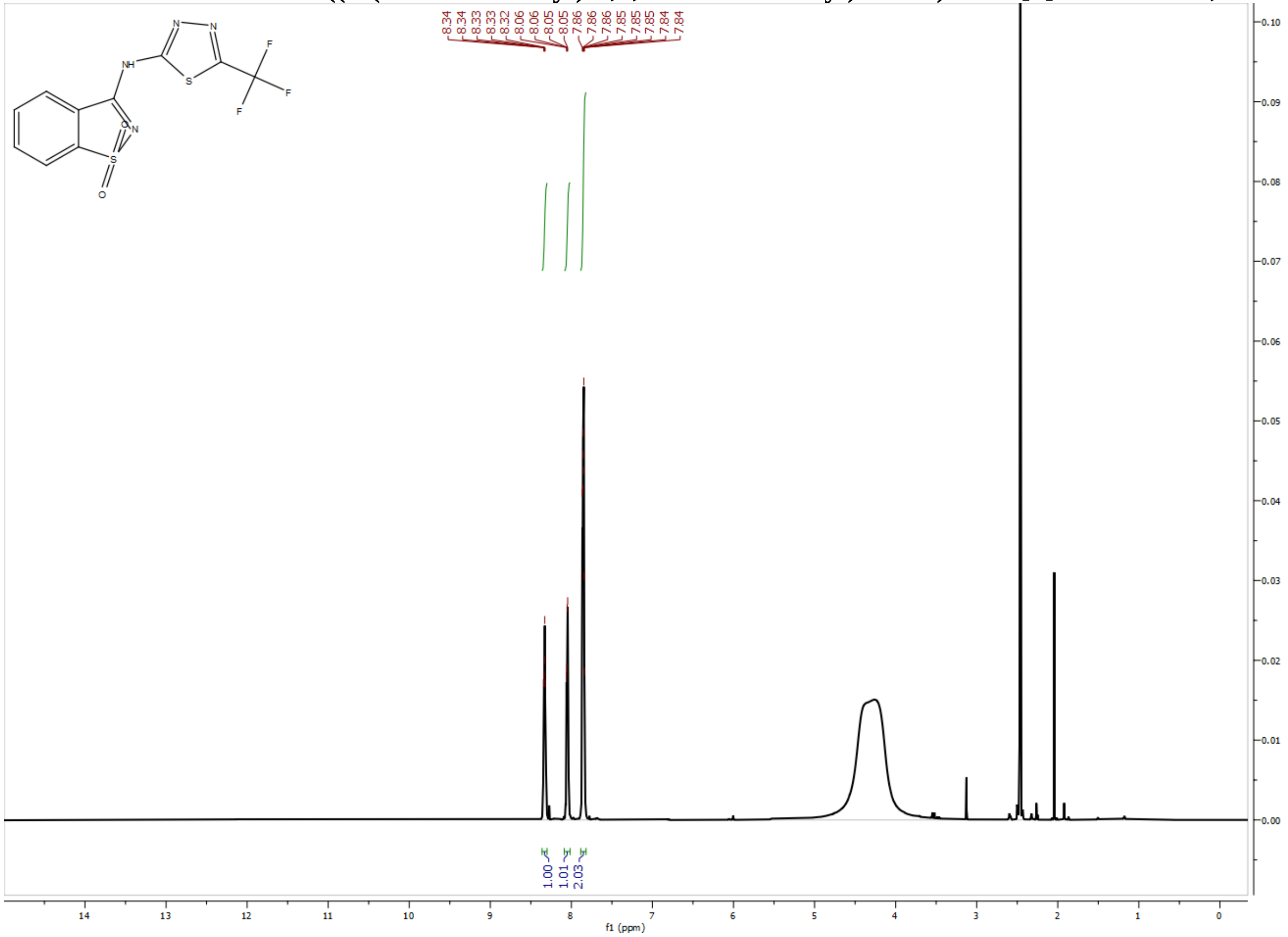

Figure S1. ^{1}H NMR spectra of 3-((5-(trifluoromethyl)-1,3,4-thiadiazol-2-yl)amino)benzo[*d*]isothiazole 1,1-dioxide (**AL14**) in DMSO-d_6.

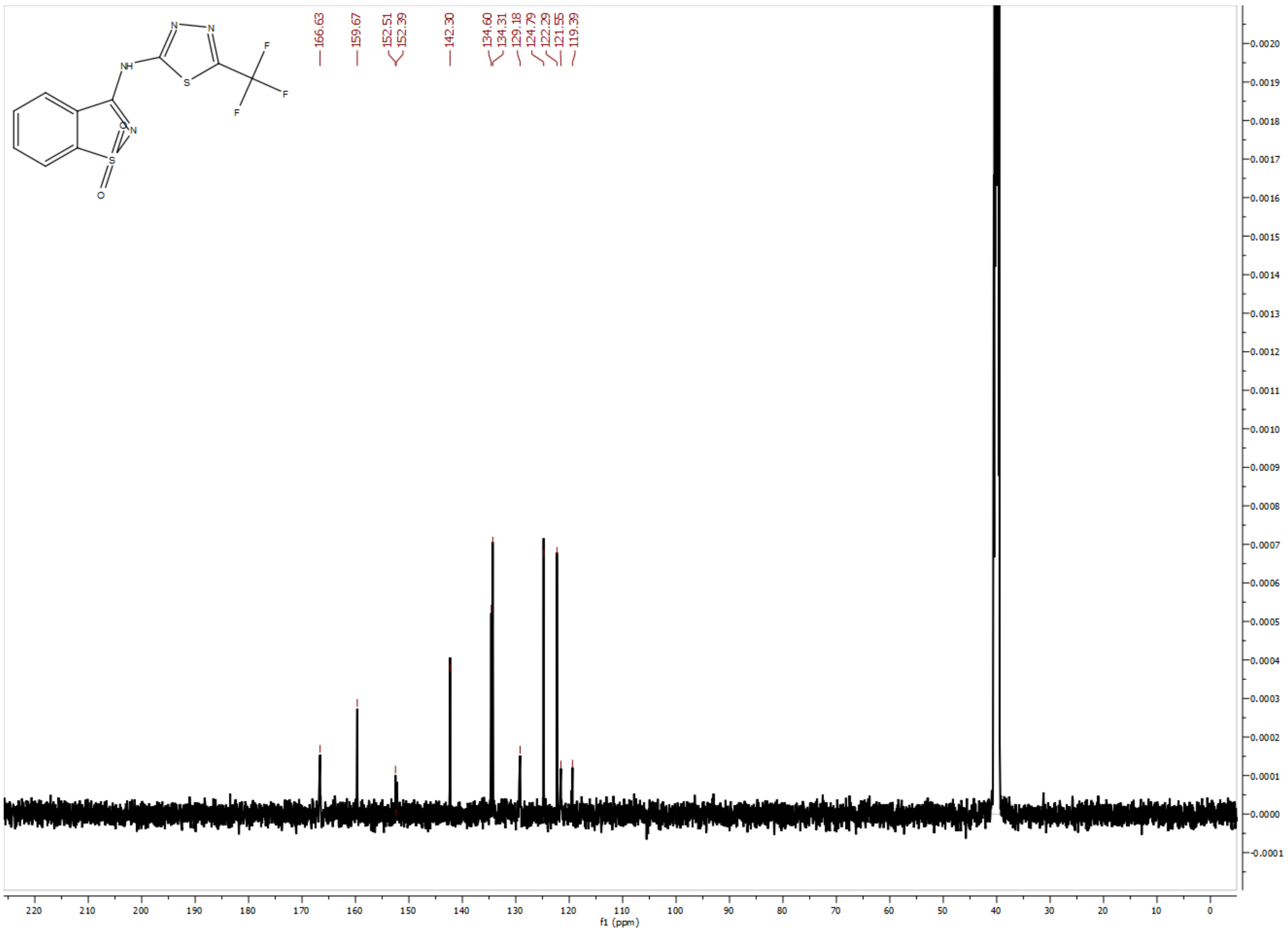

Figure S2. ^{13}C NMR spectra of 3-((5-(trifluoromethyl)-1,3,4-thiadiazol-2-yl)amino)benzo[*d*]isothiazole 1,1-dioxide (**AL14**) in DMSO-d_6.

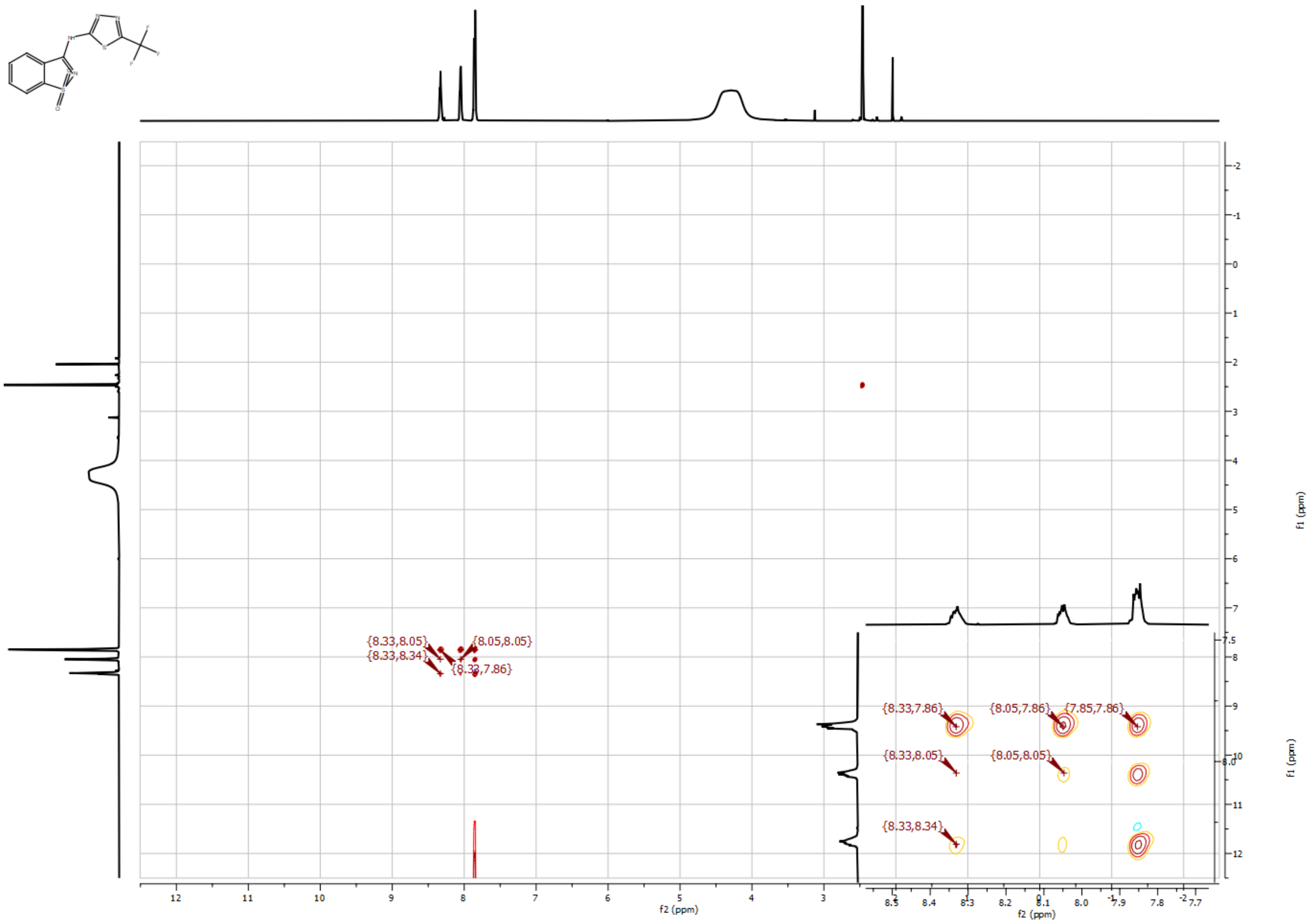

Figure S3. TOCSY spectra of 3-((5-(trifluoromethyl)-1,3,4-thiadiazol-2-yl)amino)benzo[*d*]isothiazole 1,1-dioxide (**AL14**) in DMSO-d_6.

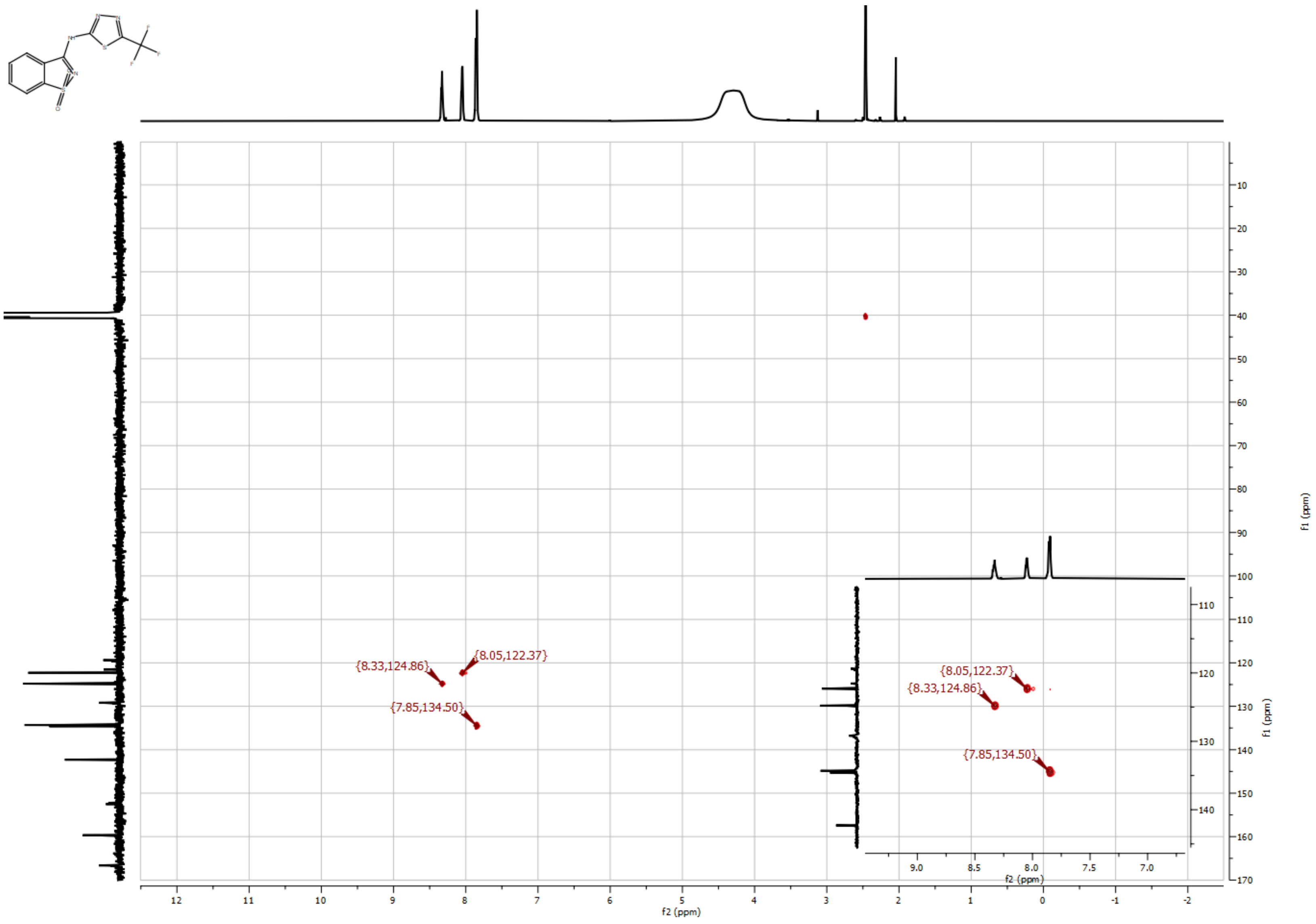

Figure S4. HSQC spectra of 3-((5-(trifluoromethyl)-1,3,4-thiadiazol-2-yl)amino)benzo[*d*]isothiazole 1,1-dioxide (**AL14**) in DMSO-d_6.

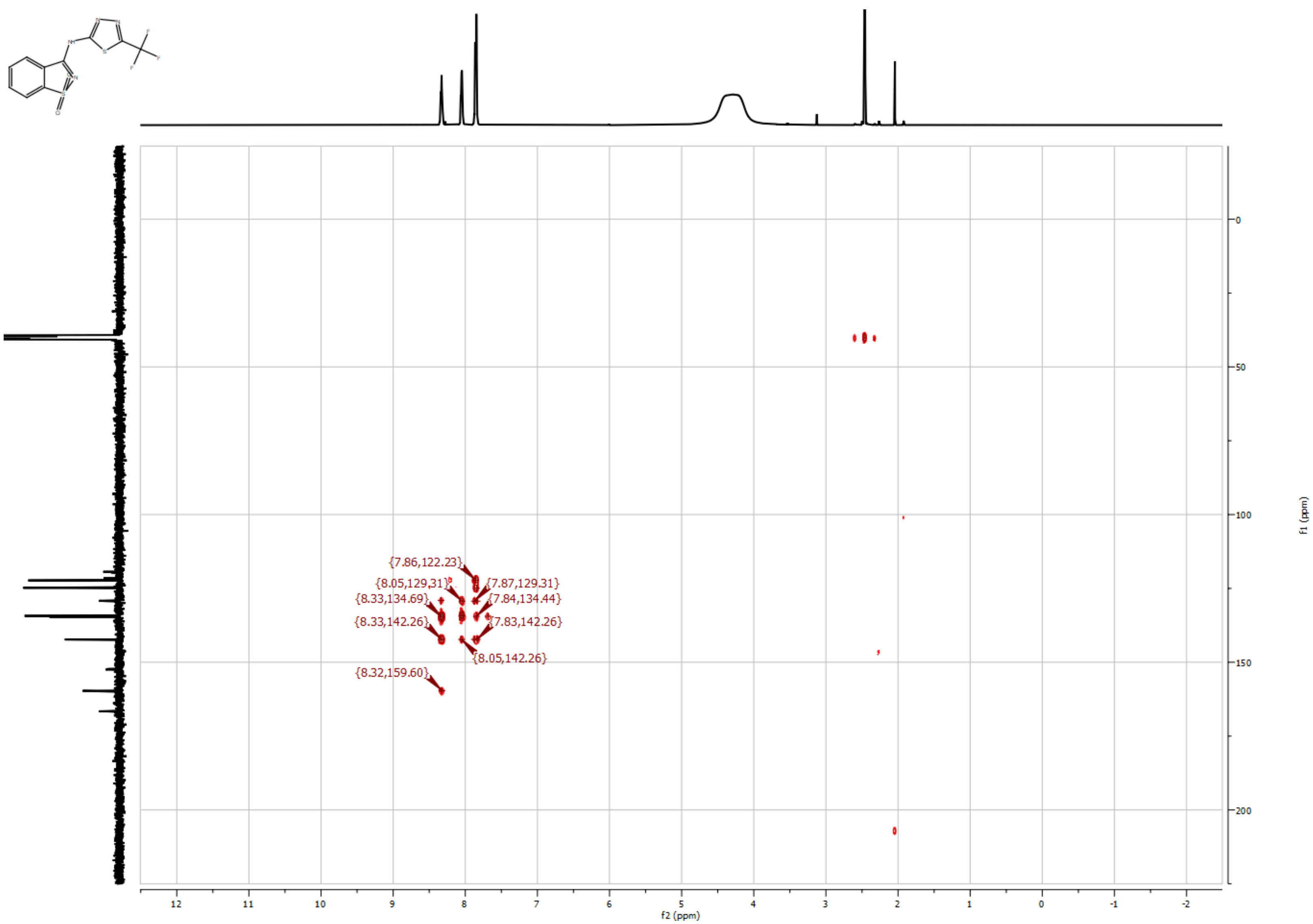

Figure S5. HMBC spectra of 3-((5-(trifluoromethyl)-1,3,4-thiadiazol-2-yl)amino)benzo[*d*]isothiazole 1,1-dioxide (**AL14**) in DMSO-d_6.

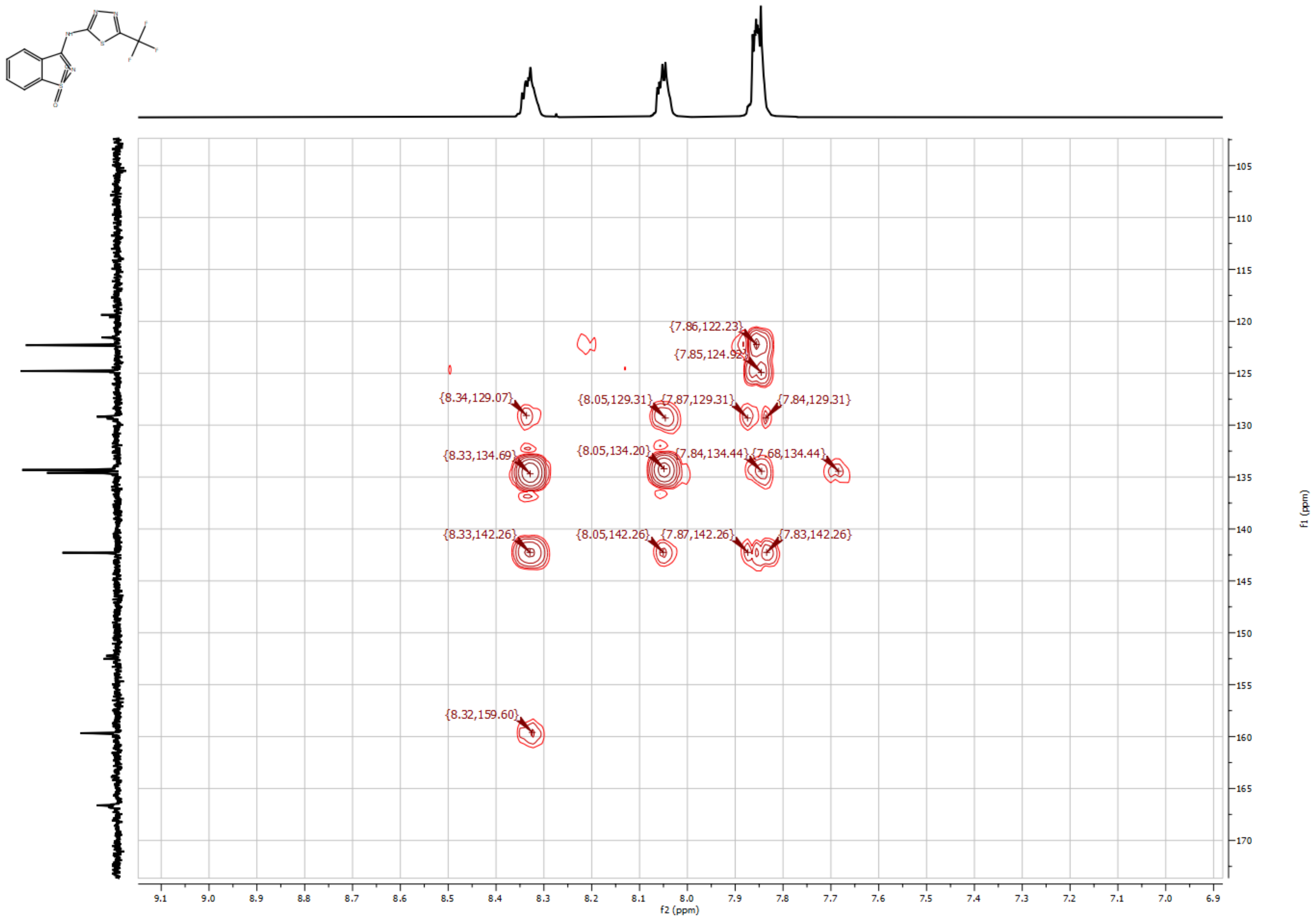

Figure S6. HMBC (expanded) spectra of 3-((5-(trifluoromethyl)-1,3,4-thiadiazol-2-yl)amino)benzo[*d*]isothiazole 1,1-dioxide (**AL14**) in DMSO-d_6.

1.2. Mass Spectra of 3-((5-(trifluoromethyl)-1,3,4-thiadiazol-2-yl)amino)benzo[*d*]isothiazole 1,1-dioxide

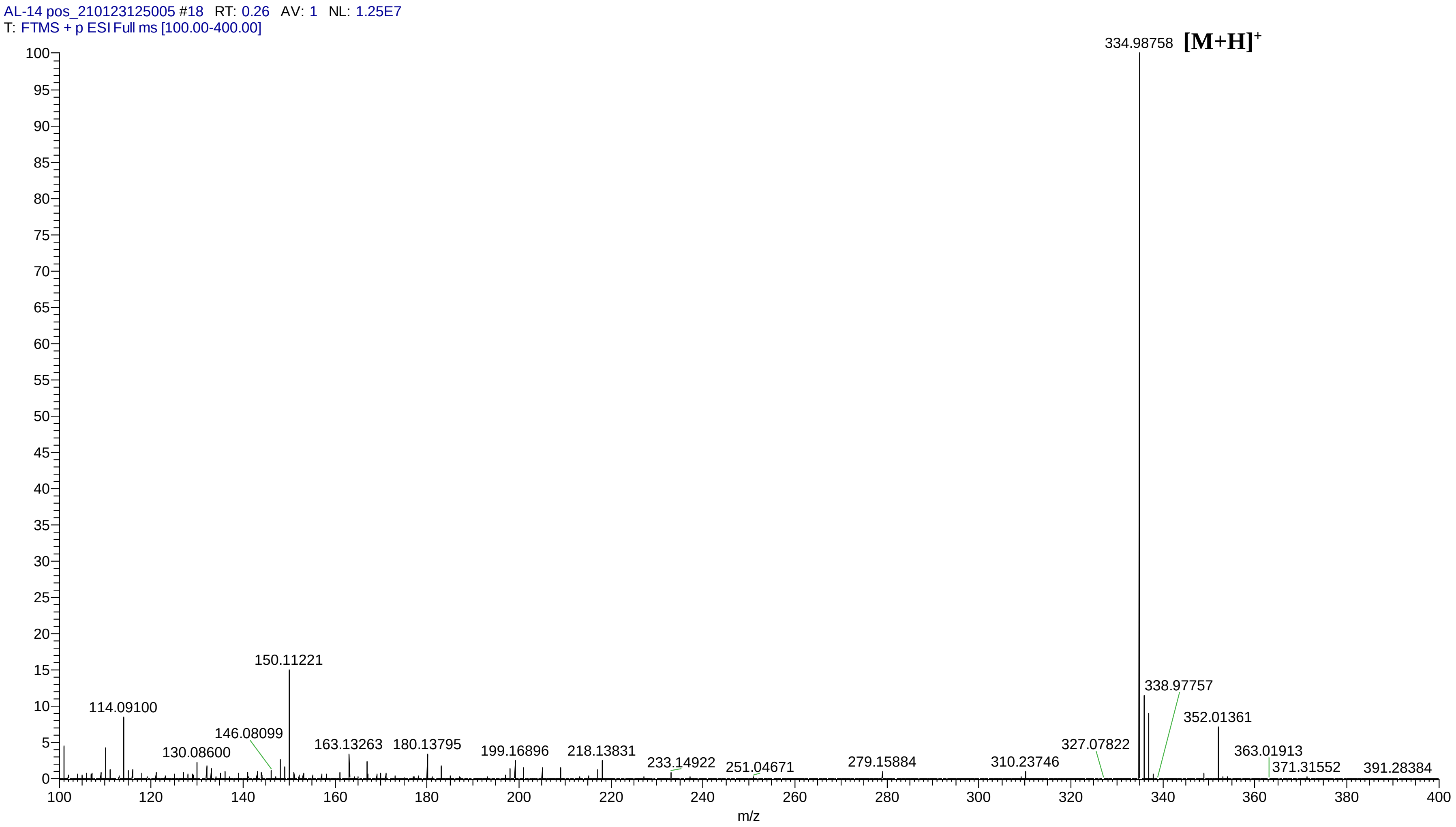

Figure S7. Electrospray ionization mass spectrum in positive-ion mode (HRMS-ESI$^+$) of 3-((5-(trifluoromethyl)-1,3,4-thiadiazol-2-yl)amino)benzo[d]isothiazole 1,1-dioxide (**AL14**).

1.3. X-ray crystallography

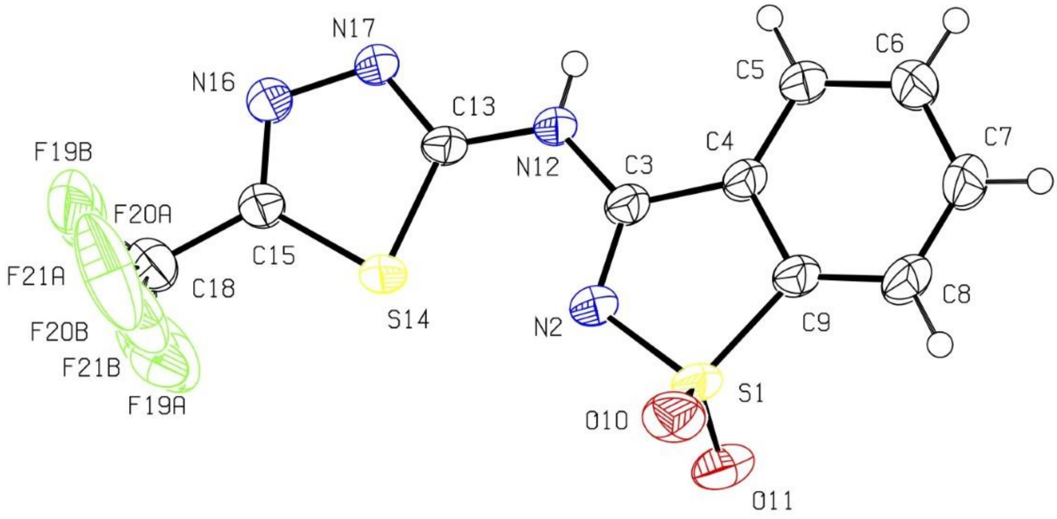

Data were collected at room temperature on an X-ray single crystal diffractometer equipped with a kappa-geometry goniometer, a 4K CCD detector (Bruker APEXII) and a sealed, fine focus, X-ray tube, emitting MoKα radiation ($\lambda = 0.71073$ Å). The *breamsstrahlung* radiation and the Kβ component were filtered by a graphite monochromator. Data collection and data reduction were performed using the Bruker APEXIII software package [S1]. The measurements were performed on a small single crystal coated with poly-fluorinated oil, mounted on top of a thin glass fibber. Initial lattice parameters were determined from a preliminary data collection consisting of set of 36 detector frames. A subsequent data collection was performed on a full sphere, using omega and phi scans of 0.5^{o} width. The data acquisition time was 10 s per frame. Reflections were merged and corrected for Lorentz and polarization effects, scan speed, and background using SAINT V8.38A [S2] included in the APEXIII package. Absorption corrections, including odd and even spherical harmonics up to rank 3 and 6, respectively, were performed using SADABS-2016/2 [S3].

Space group assignment was based upon *E* statistics, and successful refinement of the structure in space group *P*-1. The structure was solved by a dual-space method using SHELXT 2014/5 [S4] and refined by full matrix least-squares (minimizing $\Sigma w(F_o^2-F_c^2)^2$) using SHELXL-2018/1 [S5]. The weighting scheme was $w=1/[s^2(F_o^2)+(0.0466P)^2+0.2371]$ where $P=(F_o^2+2Fc^2)/3$, as suggested by SHELXL-2018/1. The final quality factors of the refinement were $R_1(I>2\sigma) = 0.0317$, $wR_{all} = 0.0406$ and GOF = 1.067 for 3097 for independent reflections and 221 refined parameters. Default SHELXL-2018/1 values for atomic scattering factors for all atoms and anomalous dispersion corrections for the non-hydrogen atoms were used. All atoms were refined anisotropically with exception for the H atoms. The H atoms attached to the C atoms were initially placed idealized positions and refined as riding using SHELXL defaults. That attached to the N12 atom had its x,y,z coordinates freely refined with an isotropic displacement parameter constrained to 1.2x of the parent atom. The F atoms of the CF3 group are disordered over two alternate positions with occupancies (0.54;0.46). The positions of the F atoms were freely refined as well as their anisotropic displacement factors without any restraints. The ORTEP plot of the molecule was generated by PLATON [S6].

CCDC 2063726 contains additional crystallographic data for this paper. These data are provided free of charge by The Cambridge Crystallographic Data Centre.

2. Experimental Methods

2.1 Synthesis

The synthesis of 3-((5-(trifluoromethyl)-1,3,4-thiadiazol-2-yl)amino)benzo[*d*]isothiazole 1,1-dioxide (**AL14**) and the preparation of pseudo-saccharyl chloride **2** were carried out by adapting the methodology described by Ismael *et al.*[S7,8], with slight modifications (Scheme S1).

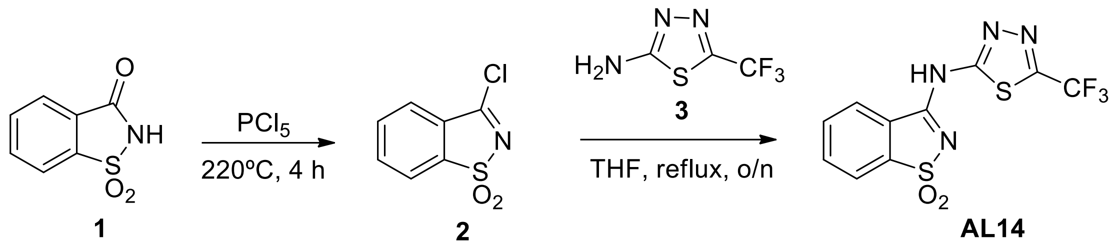

Scheme S1. Preparation of 3-((5-(trifluoromethyl)-1,3,4-thiadiazol-2-yl)amino)benzo[*d*]isothiazole 1,1-dioxide (**AL14**).

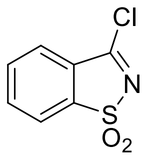

3-chloro-1,2-benzisothiazole-1,1-dioxide (2). Saccharin **1** (56 mmol) and phosphorus pentachloride (66 mmol), were heated at 220 ºC for 4 hours. On cooling to room temperature, phosphorus oxychloride was then distilled off the reaction medium, under vacuum, at 180 ºC. At 100 ºC toluene was added, and then chloroform, bellow 70 ºC, at 1:9 proportion. The mixture was allowed to crystallise at room temperature. The crystals obtained where washed with cold toluene and chloroform, giving the desired product as colourless needles (63% yield); m.p. 143-145 ºC.

^{1}H NMR (400 MHz, DMSO-d_6): δ 8.17 (d, J = 7.5 Hz, 1H), 8.00 (t, J = 8.0 Hz, 2H), 7.93 (d, J = 7.3 Hz, 1H). ^{13}C NMR (101 MHz, DMSO-d_6): δ 160.61 (s), 139.17 (s), 135.72 (s), 134.89 (s), 127.32 (s), 124.95 (s), 121.26 (s). Found: C, 41.5%; H, 2.0%; N, 6.9%; calcd for $C_7H_4NO_2SCl$: C, 41.7%; H, 2.0%; N, 7.0%. MS (EI, m/z): 201 $[M]^+$.

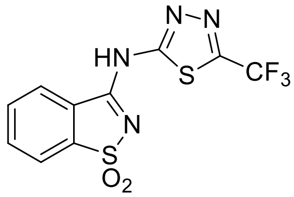

3-((5-(trifluoromethyl)-1,3,4-thiadiazol-2-yl)amino)benzo[*d*]isothiazole 1,1-dioxide (AL14).

A mixture of the corresponding *pseudo*-saccharyl chloride **2** (1.00 g, 4.96 mmol) and 2-amino-5-trifluoromethyl-1,3,4-thiadiazole **3 (**0.923 g, 5.46 mmol) in dry THF was stirred at 60 ºC for 24 h under a nitrogen atmosphere. The solvent was then evaporated under reduced pressure and the remaining solid was washed with ethyl acetate, dried under reduced pressure at room temperature. Recrystallization with ethyl acetate gave the desired compound as light green-white crystals, yield 42% (0.69 g, 2.07 mmol). mp 320ºC (dec.).

^{1}H NMR (500 MHz, DMSO-d_6): δ 8.36 – 8.30 (m, 1H), 8.08 – 8.02 (dt, J = 5.9, 2.4 Hz, 1H), 7.90 – 7.82 (m, 2H). ^{13}C NMR (126 MHz, DMSO-d_6): δ 166.63, 159.67, 152.51, 152.39, 142.30, 134.60, 134.31, 129.18, 124.79, 122.29, 121.55, 119.38. HRMS (ESI$^+$, *m/z*) calcd for $C_{10}H_6F_3N_4O_2S_2$ (M+H)$^+$: 334.98760; found 334.98758.

3. REFERENCES

(S1) Bruker, *APEX3*, Bruker AXS Inc., Madison, Wisconsin, USA. **2016**.

(S2) Bruker, *SAINT V8.38A*, Bruker AXS Inc., Madison, Wisconsin, USA. **2016**.

(S3) Krause, L., Herbst-Irmer, R., Sheldrick, G. M. and Stalke, D. Comparison of silver and molybdenum microfocus X-ray sources for single-crystal structure determination. *J. Appl. Cryst.* **2015**, *48*, 3–10, doi: 10.1107/S1600576714022985.

(S4) Sheldrick, G.M. SHELXT-Integrated Space-Group and Crystal-Structure Determination. *Acta Cryst* **2015**, *71*, 3–8, doi:10.1107/S2053273314026370.

(S5) Sheldrick, G.M. Crystal Structure Refinement with SHELXL. *Acta Crystallogr. C Struct. Chem*. **2015**, *71*, 3–8, doi:10.1107/S2053229614024218.

(S6) Spek, A. L. Structure validation in chemical crystallography, *Acta Cryst.* D **2009**, *65*, 148–155, doi: 10.1107/S090744490804362X.

(S7) Ismael, A.; Henriques, M.S.C.; Marques, C.; Rodrigues, M.; Barreira, L.; Paixao, J.A.; Fausto, R.; Cristiano, M.L.S. Exploring Saccharinate-Tetrazoles as Selective Cu(II) Ligands: Structure, Magnetic Properties and Cytotoxicity of Copper(II) Complexes Based on 5-(3-Aminosaccharyl)-Tetrazoles. *RSC Adv*. **2016**, *6*, 71628–71637, doi:10.1039/c6ra15051a.

(S8) Ismael, A.; Borba, A.; Duarte, L.; Giuliano, B.M.; Gómez-Zavaglia, A.; Cristiano, M.L.S. Structure and Photochemistry of a Novel Tetrazole-Saccharyl Conjugate Isolated in Solid Argon. *J. Mol. Struct.* **2012**, *1025*, 105–116, doi:10.1016/j.molstruc.2012.04.081.
